# Liver and spleen biometrics in childhood-onset systemic lupus erythematosus patients

**DOI:** 10.1186/1546-0096-12-S1-P327

**Published:** 2014-09-17

**Authors:** Andressa Guariento, Marco F Silva, Priscilla SF Tassetano, Sílvia M Rocha, Lúcia M Campos, Marcelo Valente, Clovis A Silva

**Affiliations:** 1 Pediatric Rheumatology Division, Faculdade De Medicina Da Universidade De São Paulo, São Paulo – SP, Brazil; 2 Pediatric Radiology Department, Faculdade De Medicina Da Universidade De São Paulo, São Paulo – SP, Brazil

## Introduction

Involvement of the reticuloendothelial system occurs in 20-50% childhood-onset systemic lupus erythematosus (c-SLE) patients at disease onset, usually associated with disease activity. Hepatomegaly and/or splenomegaly may also be associated with abnormal liver function tests. Abdominal ultrasound can be used to assess liver and spleen measurements in children and adolescents without risk of radiation. However, a systematic evaluation of these visceral organ dimensions has not been performed in c-SLE population, particularly during the disease course.

## Objectives

To evaluate liver and spleen dimensions in c-SLE patients and healthy controls and to assess possible associations between abnormalities in liver and spleen sizes with demographic data, clinical features, disease activity, cumulative damage and treatment.

## Methods

30 c-SLE patients and 30 healthy control volunteers underwent abdominal ultrasound. The following two liver measurements were performed in left hepatic lobe: craniocaudal and anteroposterior and three in right hepatic lobe (RHL): posterior craniocaudal (PCC-RHL), anterior craniocaudal and anteroposterior. Three spleen dimension measurements were also evaluated: longitudinal, transverse and anteroposterior. Demographic, clinical and laboratorial data, and treatment were assessed. Disease activity was evaluated according to SLE Disease Activity Index 2000 (SLEDAI-2K), European Consensus Lupus Activity Measurement (ECLAM) and Systemic Lupus Activity Measure (SLAM) scores.

## Results

Mean current age was similar in c-SLE and controls (170.31 ± 27.81 vs. 164.15 ± 39.25 months; p=0.486), likewise the frequency of female gender (77% vs. 63%, p=0.398). The mean of PCC-RHL dimension was significantly higher in c-SLE compared to controls (13.30 ± 1.85 vs. 12.52 ± 0.93, p=0.044). There were no differences between the other hepatic biometrics and splenic parameters (p>0.05). Further analysis in c-SLE patients according to PCC-RHL dimension > 13.3 cm (mean of this biometric measurement in 30 c-SLE patients) *versus* < 13.3 cm showed that the median of SLEDAI-2K [8 (0-18) vs. 2 (0-8), p=0.004], ECLAM [4 (0-9) vs. 2 (0-5), p=0.019] and SLAM [5 (1-13) vs. 2 (0-14), p=0.016] were significantly higher in patients with higher PCC-RHL dimension, likewise the mean of erythrocyte sedimentation rate (33.7 ± 16 vs. 22.0 ± 13 mm/1^st^ hour, p=0.038). The frequencies of nephritis were significantly higher in patients with PCC-RHL dimension > 13.3 cm *versus* < 13.3 cm (77% vs. 29%, p=0.010). The median of serum liver enzymes were similar in both groups (p>0.05). Positive correlation was observed between SLEDAI-2K and PCC-RHL (p=0.001, r=+0.595). Negative correlation was evidenced between disease duration and longitudinal dimension of spleen (p=0.031, r=**-**0.394).

## Conclusion

Our data raises the novel possibility that disease activity could lead to a subclinical and localized hepatomegaly during the disease course. Long disease duration resulted to spleen atrophy in c-SLE patients.

## Disclosure of interest

A. Guariento: None declared, M. F. Silva: None declared, P. S. F. Tassetano: None declared, S. M. Rocha: None declared, L. M. Campos: None declared, M. Valente: None declared, C. Silva Grant / Research Support from: This study was supported by Fundação de Amparo à Pesquisa do Estado de São Paulo (FAPESP - grants 2008/58238-4 to CAS), by Conselho Nacional do Desenvolvimento Científico e Tecnológico (CNPQ – grant 302724/2011-7 to CAS), by Federico Foundation to CAS and by Núcleo de Apoio à Pesquisa “Saúde da Criança e do Adolescente” da USP (NAP-CriAd).

